# Measles-like hypermutation of a Newcastle disease virus drives fatal encephalitis during BCMA × CD3 T-cell-redirecting immunotherapy

**DOI:** 10.1007/s00401-026-03116-0

**Published:** 2026-11-11

**Authors:** Christina Krueger, Christoph Schaefers, Thomas Guenther, Lisa Leypoldt, Ricardo Ahrens, Winfried Alsdorf, Maximilian Al-Bazaz, Katja Weisel, Sofie-Katrin Kadel, Leo Rasche, Niels Weinhold, Marc Raab, Julia Hoppe, Alexander Postel, Denise Heim, Kerstin Reumann, Alexis Robitaille, Patrick Bluemke, Felix Fischbach, Eckhard Schlemm, Manuel Friese, Tim Magnus, Axel Nierhaus, Stefan Kluge, Benjamin Ondruschka, Catena Kresbach, Matthias Dottermusch, Marc Luetgehetmann, Nicole Fischer, Adam Grundhoff

**Affiliations:** 1 Department of Neurology, University Medical Center Hamburg-Eppendorf, Hamburg, Germany; 2 Department of Oncology, Hematology and Bone Marrow Transplantation with the Section Pneumology, University Medical Center Hamburg-Eppendorf, Hamburg, Germany; 3 Leibniz Institute of Virology, Martinistr. 52, 20252 Hamburg, Germany; 4 Department of Internal Medicine II, University Hospital Würzburg, Würzburg, Germany; 5 Department of Hematology, Oncology and Rheumatology, Heidelberg University Hospital, Heidelberg, Germany; 6 Institute of Virology, Stiftung Tiermedizinische Hochschule Hannover, Hanover, Germany; 7 Institute of Medical Microbiology, Virology and Hygiene, University Medical Center Hamburg-Eppendorf, Hamburg, Germany; 8 Institute for Molecular Virology and Tumorvirology, University Medical Center Hamburg-Eppendorf, Martinistr. 46, 20246 Hamburg, Germany; 9 Department of Intensive Care, University Medical Center Hamburg-Eppendorf, Hamburg, Germany; 10 Institute of Legal Medicine, University Medical Center Hamburg-Eppendorf, Hamburg, Germany; 11 Institute of Neuropathology, University Medical Center Hamburg-Eppendorf, Hamburg, Germany

**Keywords:** Avian orthoavulavirus-1, Newcastle disease virus, Neuroinvasive encephalitis, BCMA × CD3 bispecific antibody immunotherapy, Metagenomic next-generation sequencing (mNGS), ADAR-mediated RNA editing, Within-host viral evolution, Persistent paramyxovirus infection

## Abstract

Newcastle disease virus (NDV or AOaV-1) is an avian paramyxovirus that typically causes only mild, self-limiting disease in humans. Here, we report fatal neuroinvasive AOaV-1 infection in a patient with multiple myeloma receiving maintenance therapy with teclistamab, daratumumab, and lenalidomide following frontline teclistamab-based treatment and autologous stem cell transplantation. Following rapidly progressive brainstem–cerebellar encephalitis and negative conventional microbiological testing, unbiased metagenomic RNA sequencing of a cerebellar biopsy identified AOaV-1 at high intraparenchymal abundance. Viral genome analysis revealed striking A-to-G and U-to-C substitutions selectively concentrated in the matrix gene, consistent with ADAR-mediated RNA editing. Long-read sequencing resolved two genetically distinct, independently hypermutated CNS haplotypes. Post-mortem analysis demonstrated widespread dissemination and continued viral diversification in multiple brain regions, whereas a peripheral urine sample contained a minimally mutated putative ancestral lineage. These findings support peripheral infection followed by CNS seeding and compartmentalized within-host evolution. The selective matrix-gene hypermutation closely parallels persistent measles virus infection in subacute sclerosing panencephalitis, revealing a shared mechanism of paramyxovirus adaptation to the CNS. Profound immunotherapy-associated immune dysfunction may thus create niches enabling normally low-pathogenic zoonotic viruses to persist, evolve, and acquire severe neurovirulence.

**Supplementary Information:**

The online version contains supplementary material available at https://doi.org/10.1007/s00401-026-03116-0.

## Introduction

ADAR-mediated hypermutation of the viral matrix (M) gene is a defining feature of persistent measles virus infection in subacute sclerosing panencephalitis (SSPE), where RNA editing impairs viral assembly, promotes cell-to-cell spread, and facilitates long-term persistence within neural tissue [5, 14]. Similar matrix-gene hypermutation has been described in a single case of chronic encephalitis caused by vaccine-derived mumps virus [12], suggesting that host-mediated RNA editing may represent a shared mechanism of persistent paramyxovirus infection. Whether comparable evolutionary mechanisms occur in other paramyxovirus infections, however, has remained unknown. Avian orthoavulavirus-1 (AOaV-1), commonly known as Newcastle disease virus (NDV), is a highly contagious paramyxovirus that infects poultry and wild birds worldwide. Infection causes severe respiratory, neurologic and gastrointestinal disease in infected birds, with mortality rates that can reach 100% in unvaccinated flocks. While AOaV-1 can be transmitted from birds to humans, clinical symptoms of zoonotic infection are usually restricted to mild, self-limiting conjunctivitis. Here, we describe a fatal neuroinvasive infection with AOaV-1 during BCMA × CD3 T-cell-redirecting immunotherapy for multiple myeloma. We demonstrate selective ADAR-mediated matrix-gene hypermutation, compartmentalized CNS evolution, and within-host viral diversification, closely paralleling the molecular evolution observed in persistent measles virus infection. Our data highlight a previously unrecognized risk of therapy-associated viral evolution of low-pathogenic zoonotic paramyxoviruses leading to the emergence of highly neurovirulent variants.

## Material and methods

### Consent

Post-mortem examination, including neuropathological and virological analyses, was performed with the consent of the patient’s legally authorized representative and in accordance with local institutional and legal requirements.

### Reverse transcription (RT-)PCR

Quantitative diagnostic RT-PCR for Newcastle disease virus was performed by targeting the viral M and F genes using previously published primers and methods [2, 8]. For long-read sequencing, reverse transcription was carried out with a primer targeting the negative (genomic polarity) strand of NDV, followed by long-range PCR amplification of nucleotides 1984–5217 of the viral genome. Primer and probe sequences, as well as details on cDNA synthesis and amplification protocols are provided in the supplementary methods.

### Metagenomic analysis

**Fig. 1:**
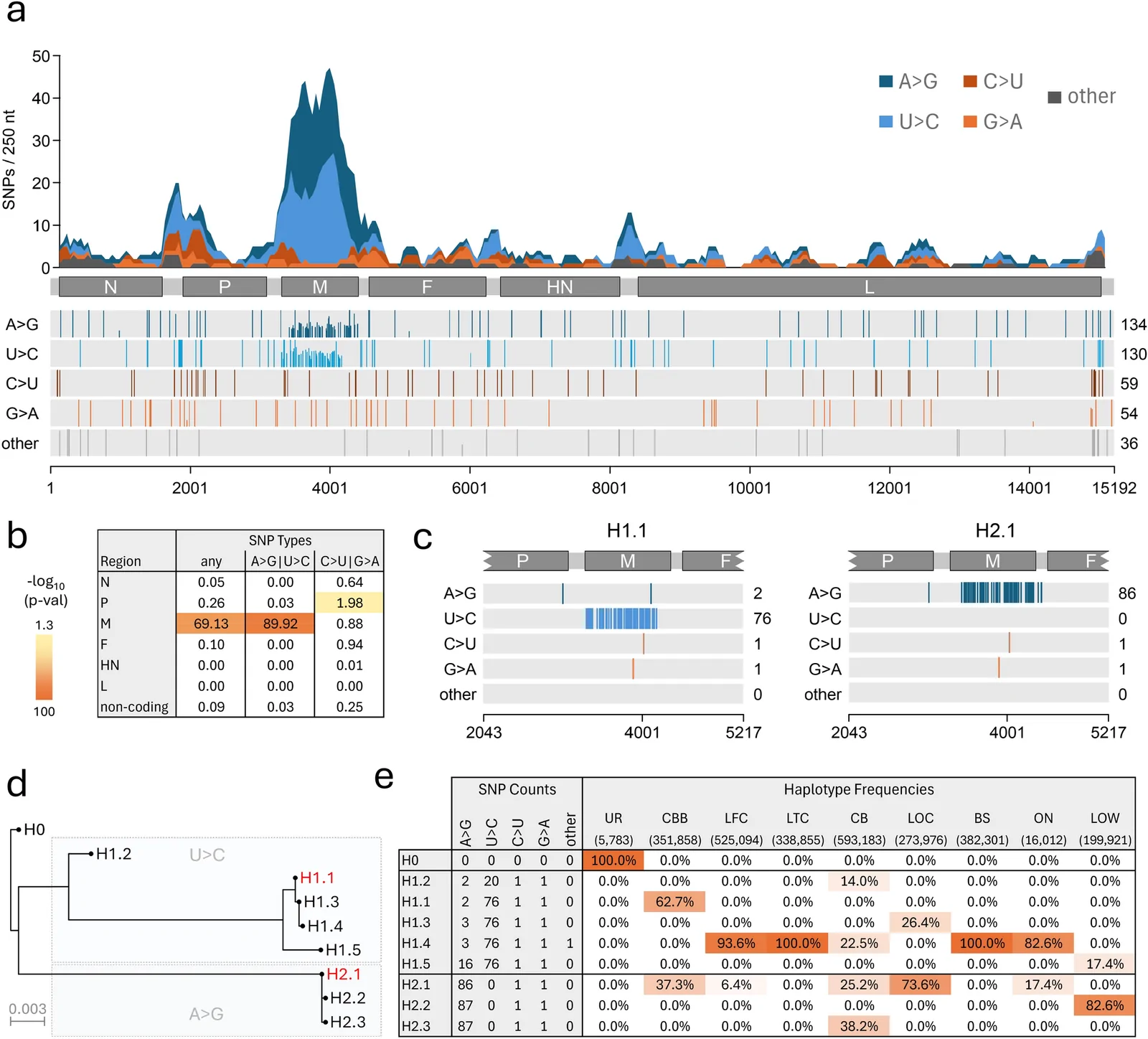
Genomic analysis of AOaV-1 SMP60/HH/2025. **a** Genome-wide distribution of single nucleotide polymorphisms (SNPs) in metagenomic reads mapped to Newcastle disease virus isolate DOZA007M22. Upper panel: cumulative SNP counts by substitution type in 250nt windows. Lower panels: genomic position, relative frequency (height of bars; full height = 100%) and total counts of individual substitutions. **b** Statistical significance (hypergeometric test, − log10 *P* values) of regional enrichment of all SNPs and SNPs that may result from ADAR (A > G, U > C) or APOPEC (C > U, G > A) editing events. **c** Nucleotide substitutions in cerebellar biopsy haplotypes H1.1 and H2.1 relative to the ancestral urine-derived haplotype H0. **d** Maximum-likelihood phylogeny of haplotypes recovered by amplicon sequencing from urine, cerebellar biopsy, and post-mortem samples. **e** Distribution of viral haplotypes across urine (UR), cerebellar biopsy (CBB), and post-mortem CNS regions: left frontal cortex (LFC), left temporal cortex (LTC), cerebellum (CB), left occipital cortex (LOC), brainstem (BS), optic nerve (ON), and left occipital white matter (LOW). Percentages indicate the proportion of amplicon reads represented by each haplotype. Total reads per sample are shown below sample labels; SNP counts relative to H0 are shown to the left of each haplotype

Untargeted high-throughput short-read sequencing was performed on stranded libraries prepared from total RNA isolated from clinical samples. Libraries were sequenced on Element Biosciences AVITI and Illumina NextSeq 2000 instruments. Resulting data were analyzed as recently described using an in-house pathogen detection pipeline combining mapping, kmer, and de novo assembly approaches [1, 9]. For identification and abundance estimation of SNPs, metagenomic reads were mapped to AOaV-1 isolate DOZA007M22 (GenBank accession OQ745938), followed by SNP detection using bcftools (v1.17). Resulting vcf files were filtered for variant calls with quality scores above 50 for the analysis of major and minor variants shown in Fig. 1A. For metagenomic consensus sequences, variant calls were further filtered for those with allele frequencies of at least 0.9. Detailed information on sample preparation, sequencing and bioinformatic analysis is provided in the supplementary methods.

### Long-read sequencing and haplotype analysis

Long-range PCR amplicons were subjected to end repair, dA-tailing and ligation of sequencing adapters, followed by sequencing on an Oxford Nanopore Technologies (ONT) PromethION 2 Solo device. Reads were demultiplexed and quality-filtered prior to downstream haplotype reconstruction and abundance estimation with strainline [10]. Phylogenetic relationships were inferred using maximum-likelihood based on a general time-reversible nucleotide substitution model with gamma-distributed rate variation. Detailed information on long-read sequencing, haplotype reconstruction and phylogenetic analysis can be found in supplementary methods.

### Statistical analysis of SNP distribution

The probability of observed SNPs of a given type in specific subgenomic regions exceeding those expected by chance was calculated using the hypergeometric test function phyper in R (v4.5.2) with the following parameters: *q* = observed number of SNPs of type *t* in region of interest, *m* = total number of SNPs of type *t* in the genome, *n* = genome length − *m*, *k* = length of the region of interest.

## Results and discussion

Teclistamab, a BCMA × CD3 bispecific T-cell engager, induces deep and durable responses in multiple myeloma but is associated with profound humoral and cellular immune dysfunction, predisposing patients to severe forms of opportunistic infections [6, 11]. A 70-year-old patient with multiple myeloma, receiving maintenance therapy with teclistamab, daratumumab, and lenalidomide after frontline teclistamab-based treatment within the phase II MajesTEC-5 trial [13] presented with acute diplopia and gait instability, rapidly progressing to a brainstem–cerebellar syndrome with ocular motor deficits, nystagmus, truncal and limb ataxia, and progressive motor impairment, subsequently advancing to bulbar dysfunction, tetraparesis, and impaired consciousness. The patient had frequently traveled to southern Africa, most recently in February 2025. Despite fulminant clinical and radiological progression, repeated cerebrospinal fluid analyses revealed only nonspecific inflammatory changes, and extensive microbiological testing, including broad PCR-based pathogen screening, remained negative (Supplementary Table S1). Given the rapidly progressive encephalitic syndrome in the setting of profound therapy-associated immune dysfunction, stereotactic cerebellar biopsy was performed. Unbiased metagenomic short-read RNA sequencing identified an avian orthoavulavirus-1 (designated AOaV-1 SMP60/HH/2025) as the sole pathogen, establishing the diagnosis of neuroinvasive AOaV-1 infection (Fig. 1A, Supplementary Tables S2, S3). Approximately 1% of sequencing reads mapped to the viral genome, indicating an exceptionally high intraparenchymal viral burden, confirmed by quantitative RT-PCR (Supplementary Table S1). Retrospective testing of clinical specimens collected before and after biopsy detected AOaV-1 RNA in a single urine sample obtained at day 27 (Supplementary Table S1), suggesting low-level shedding from residual peripheral infection reservoirs. Histopathological examination of the cerebellar biopsy showed preserved architecture with leptomeningeal and parenchymal T-cell infiltration, most prominent around vessels, accompanied by diffuse microglial activation and reactive astrocytosis (Fig. S1A–E). No overt destructive lesion was identified in the biopsy.

Variant calling among metagenomic short reads mapped to the most closely related NDV reference sequence (Supplementary Fig. S2) revealed a striking accumulation (− log_10_ *p*-value 89.92) of A-to-G and U-to-C substitutions, the characteristic mutational signature of ADAR-mediated RNA editing (Fig. 1A, B; Supplementary Fig. S3) in the viral matrix gene. Evaluation of mutation frequencies (bar plots in Fig. 1A) furthermore suggested the presence of at least two closely related viral CNS-resident populations with distinct ADAR-associated mutation signatures. The moderate enrichment of C-to-U/G-to-A substitutions in the P gene, in contrast, was considered likely to reflect natural sequence variation relative to the reference sequence.

To confirm our hypothesis, we performed long-read sequencing of amplicons containing the M gene and partial P and F genes from the brain biopsy and urine samples. Haplotype reconstruction from the urine sample recovered a single, minimally mutated haplotype (designated H0), likely representing the original, fully infectious virus. In contrast, analysis of the biopsy sample resolved two genetically distinct haplotypes harboring almost exclusively either U-to-C (haplotype H1.1) or A-to-G (haplotype H2.1) hypermutation patterns (Fig. 1C; Supplementary Figs. S4 and S5), consistent with extensive ADAR-mediated A-to-I editing of negative-strand (genome) or positive-strand (antigenome) RNA substrates, respectively. The strikingly selective hypermutation patterns observed in AOaV-1 closely parallel the molecular signatures of persistent measles virus infection in subacute sclerosing panencephalitis and chronic mumps virus encephalitis [3, 4, 12, 14], suggesting common mechanisms of host-driven paramyxovirus evolution toward virulent variants in the CNS.

Following metagenomic diagnosis, off-label remdesivir treatment was initiated because of its presumed antiviral activity and central nervous system penetration. Despite antiviral therapy and intensive supportive care, neurological deterioration progressed to deep coma with complete loss of brainstem reflexes. In accordance with the patient's advance directive, life-sustaining treatment was withdrawn, and the patient died shortly thereafter. Evaluation of multiple post-mortem cerebellar sections showed lymphocytic inflammation and glial reactivity in a distribution broadly similar to that in the biopsy (Supplementary Fig. 1F–J). Lymphocytic inflammation and glial activation were identified in all histologically evaluable post-mortem CNS regions with detectable AOaV-1 RNA (Supplementary Table S1). Autolysis limited assessment of neuronal loss and other antemortem tissue changes.

We performed long-read haplotype reconstruction from multiple post-mortem brain regions and compared the sequences to those previously recovered from the biopsy and urine samples. As shown in Fig. 1D, E, and Supplementary Figs. S4, S5, phylogenetic analysis demonstrated widespread dissemination of H1.1 and H2.1-related lineages in the CNS. Inspection of substitution patterns suggests that four major episodic editing events gave rise to an equal number of mutated haplotype groups (main branches in Fig. 1D), with a small number of additional substitutions creating minor diversity among some of the groups. Our findings therefore support a stepwise model in which peripheral AOaV-1 infection had seeded the CNS, followed by compartmentalized, within-host viral evolution driven by recurrent ADAR-mediated RNA editing. This evolutionary trajectory suggests that host-driven evolution of hypermutated, highly neurovirulent paramyxovirus variants is not limited to human measles and mumps viruses [3, 5, 12], but can also occur in zoonotic paramyxovirus infections that are normally considered low pathogenic. Interestingly, a recent study reported similar hypermutations in attenuated Newcastle disease virus vectors developed as vaccine candidates [7].

Although this study represents a single case, our data suggest that profound therapy-associated immune dysregulation may facilitate prolonged AOaV-1 persistence and within-host evolution. Given the rapidly expanding use of T-cell-redirecting immunotherapies and recurrent outbreaks of AOaV-1 in bird populations worldwide, these potential risks warrant consideration in patient management and infectious disease surveillance. More broadly, therapy-induced immunological niches permissive to viral persistence and evolution may represent an underappreciated driver of severe infectious disease. In such settings, unbiased metagenomic sequencing may be particularly valuable for identifying unexpected or highly divergent pathogens that escape conventional diagnostic testing.

## Supplementary Information

Below is the link to the electronic supplementary material.

Supplementary file1 (PDF 5439 KB)

## Data Availability

All relevant short-read and long-read sequencing data have been deposited in the European Nucleotide Archive (ENA) under accession PRJEB111208. Annotated haplotype genome sequences reconstructed from short and long read sequencing data (see supplementary methods) have been deposited in GenBank under accession numbers QB284243 (H0), QB284244 (H1.1), QB284245 (H1.2), QB284246 (H1.3), QB284247 (H1.4), QB284248 (H1.5), QB284249 (H2.1), QB284250 (H2.2), and QB284251 (H2.3).
