# Supplementary material for: Measles-like hypermutation of a Newcastle disease virus drives fatal encephalitis during BCMA × CD3 T-cell-redirecting immunotherapy
Source: Acta Neuropathol. 2026 Nov 11;152(1):68. doi: 10.1007/s00401-026-03116-0 (PMC13754954; doi:10.1007/s00401-026-03116-0)
Supplement: Supplementary file 1 [file 401_2026_3116_MOESM1_ESM.pdf]

Measles-like hypermutation of a Newcastle disease virus drives fatal encephalitis during BCMA × CD3 T-cell–redirecting immunotherapy

Christina Krüger[1]*, Christoph Schaefers[2*], Thomas Günther[3*], Lisa Leypoldt[2], Ricardo Ahrens[2], Winfried Alsdorf[2], Maximilian Al-Bazaz[2], Katja Weisel[2], Sofie-Katrin Kadel[4], Leo Rasche[4], Niels Weinhold[5], Marc Raab[5], Julia Hoppe[1], Alexander Postel[6], Denise Heim[7], Kerstin Reumann[3], Alexis Robitaille[3,8], Patrick Blümke[3], Felix Fischbach[1], Eckhard Schlemm[1], Manuel Friese[1], Tim Magnus[1], Axel Nierhaus[9], Stefan Kluge[9], Benjamin Ondruschka[10], Catena Kresbach[11], Matthias Dottermusch[11], Marc Lütgehetmann[7#], Nicole Fischer[8#], Adam Grundhoff[3#]

Supplementary Information:

1. *Detailed neuropathological presentation and diagnostic evaluation*
2. *Supplementary haplotype information*
3. *Supplementary methods*
4. *Supplementary tables S1-S3*
5. *Supplementary Figures S1-S6*
6. *References*
7. *Other information*

1. Detailed neurological presentation and diagnostic evaluation

A 70-year-old patient with IgG lambda multiple myeloma, diagnosed in 2022, was admitted in July 2025 with acute-onset diplopia and gait disturbance. Neurological examination revealed bilateral eye abduction deficits, upbeat nystagmus, truncal and limb ataxia, and left-sided lower-limb paresis.

Regarding multiple myeloma, the patient had received frontline quadruplet induction therapy with teclistamab, daratumumab, lenalidomide, and dexamethasone, followed by high-dose melphalan and autologous stem cell transplantation. The patient was enrolled in the phase II GMMG-HD10/DSMM-

XX (MajesTEC-5) trial evaluating teclistamab-based induction therapy for transplant-eligible newly diagnosed multiple myeloma [11]. Following induction and autologous stem cell transplantation, maintenance treatment with teclistamab, daratumumab, and lenalidomide was continued according to the study protocol. At the time of presentation, the patient had achieved a deep and durable response with measurable residual disease–negative complete remission. Prophylactic therapy included monthly intravenous immunoglobulin replacement, acyclovir, and cotrimoxazole. No other relevant comorbidities were reported. The patient's travel history revealed frequent stays in southern Africa, most recently in February 2025, indicating potential exposure to infectious agents not endemic to the patient's place of residence or hospitalization in northern Germany.

Laboratory investigations at admission demonstrated profound treatment-related immune dysfunction. Peripheral B cells were undetectable (0% of lymphocytes; absolute count 0 cells/µL), consistent with sustained BCMA-directed therapy and profound impairment of humoral immunity.

Neurological function deteriorated rapidly over the following days, with progressive dysarthria, worsening ataxia and tetraparesis. Bilateral facial palsy emerged later in the clinical course and was considered nuclear rather than peripheral, consistent with evolving brainstem pathology on imaging. Approximately one week after admission, the patient experienced a sudden decline in consciousness with loss of protective airway reflexes, necessitating transfer to the intensive care unit and endotracheal intubation.

Brain magnetic resonance imaging revealed progressive fluid-attenuated inversion recovery (FLAIR) hyperintensities involving the brainstem, cerebellum, and bilateral thalami. There were no radiological features suggestive of vascular disease, malignant infiltration, or demyelination.

Initial cerebrospinal fluid (CSF) analysis showed mild pleocytosis (6 leukocytes/µL), which increased to 62 cells/µL on subsequent examinations, accompanied by rising protein concentrations ranging from 570 to 1005 mg/L and elevated lactate levels between 1.93 and 3.1 mmol/L. Serial CSF glucose concentrations remained normal throughout. Repeated CSF cultures were sterile, and comprehensive microbiological investigations, including broad PCR-based testing for viral, bacterial, fungal, and

parasitic pathogens failed to identify an infectious etiology (suppl. Table 1). Serial CSF cytology and neuropathological assessments showed no evidence of malignant cells. Comprehensive testing for autoimmune and paraneoplastic antibodies was also negative.

Electroencephalography initially demonstrated moderate generalized slowing (theta rhythm) with a bifrontal delta focus, without epileptiform activity. With clinical progression, Electroencephalography (EEG) findings evolved to severe generalized slowing (delta and subdelta), consistent with diffuse cerebral dysfunction. Additionally, high-amplitude monomorphic delta activity was detected frontally. No periodic, polymorph complexes consisting of sharp and slow waves discharges typically seen in subacute sclerosing panencephalitis (SSPE) were found.

Given the relentless neurological deterioration, profound combined humoral and cellular immunodeficiency, and persistently negative conventional diagnostic investigations (Supplementary Table S1), a multidisciplinary decision was made to pursue stereotactic cerebellar biopsy. Histopathological examination revealed a lymphocytic meningoencephalitis with perivascular and intraparenchymal infiltrates predominantly composed of $CD3^+$ and $CD8^+$ T cells, accompanied by microglial activation. There was no evidence of malignant infiltration or significant B-cell involvement. Despite broad-spectrum antimicrobial therapy and escalating immunomodulatory treatment for presumed autoimmune encephalitis, neurological deterioration progressed to coma, respiratory failure and death.

2. Supplementary haplotype information

Viral haplotypes were reconstructed by performed by long-read sequencing of amplicons covering the M gene and partial sequences of the flanking P and F genes (nucleotides. 1984-5217 of the viral genome). cDNA synthesis was performed with a primer targeting the negative strand of the viral genome to ensure capture of genomic RNA. Viral haplotype sequencing was performed from the urine sample collected on day 27 after admission, the brain biopsy sample collected on day 21, and the CNS post-mortem tissues collected on day 41 (Table S1). The urine sample was included because it had

tested weakly positive by PCR and we suspected that it might harbor sequences of the original, fully infectious virus. Indeed, the single haplotype H0 recovered from the urine sample was phylogenetically ancestral to CNS-resident specimen. It harbored a substantially lower mutational burden compared to CNS-derived specimen and did not show any enrichment of mutations in the viral Matrix gene (see Figures S4 for alignments of AOaV-1 SMP60/HH/2025 haplotypes to the closest relative DOZA007M22, and Figure S5 for a within-sample alignment to haplotype H0). Compared to H0, the prototypical haplotypes H1.1 and H2.1 exhibited almost exclusively U-to-C or A-to-G mutations, respectively, resulting in 47 or 54 non-synonymous codon changes in the region encoding the matrix protein (Supplementary Figure S6). In measles virus infection, disruption of matrix protein function impairs virion assembly and favors direct cell-to-cell spread via membrane fusion a mode of propagation that enhances persistence within neural tissue, reduces susceptibility to antibody-mediated neutralization, and contributes to chronic progressive CNS disease [4, 10]. The selective enrichment of hypermutations within the matrix gene therefore provides a plausible mechanistic link between host RNA editing, viral persistence and neurovirulence.

Most sequences recovered from post-mortem CNS samples were identical or highly similar to haplotypes H1.1 and H2.1, with individual samples from CNS regions harboring different frequencies of H1- or H2-related haplotypes. More distantly related sequences comprised haplotypes H1.2 and H1.5, recovered from cerebellar and left occipital white matter at frequencies of approximately 14% and 17%, respectively (see Figure 1E in main manuscript).

The recovery of both ancestral and hypermutated viral haplotypes across anatomical compartments provides direct evidence of compartmentalized within-host evolution. The segregation of distinct mutation spectra across central nervous system–derived haplotypes, together with the limited presence of intermediate variants, suggests that ADAR-mediated editing may have occurred in episodic bursts during viral transcription and replication, rather than through gradual accumulation of mutations. Such punctuated editing events could accelerate viral diversification and facilitate the rapid emergence of phenotypically distinct quasi-species under conditions of impaired immune surveillance.

3. Supplementary methods

RNA extraction

Nucleic acid extraction from liquid specimens (urine, cerebrospinal fluid, and plasma) was performed using the MagNA Pure 96 system (Roche Molecular Systems, CA, USA).

Human tissue samples were processed in 2 mL microtubes prefilled with ceramic beads (Precellys Lysing Kit; Bertin Technologies SAS, Montigny-le-Bretonneux, France) and 1 mL phosphate-buffered saline. Tissue homogenization was performed using the Precellys 24 Tissue Homogenizer (Bertin Technologies SAS). For nucleic acid extraction, 200 µL of the resulting tissue lysate was transferred to the MagNA Pure 96 system and processed using the Viral NA Small Volume Kit (Roche Molecular Systems), following the manufacturer's recommendations.

RNA concentration and integrity were assessed using the Agilent TapeStation system according to standard quality control procedures.

NDV diagnostic PCR

Reverse transcription PCR (RT-PCR) was performed for the detection of the Newcastle disease virus (NDV) RNA genome using a previously published method [2]. Amplification targeted the matrix (M) gene using primers NDV_M-4100_Fw (5'-AGTGATGTGCTCGGACCTTC-3') and NDV_M-4220_Rev (5'-CCTGAGGAGAGGCATTTGCTA-3'), together with the hydrolysis probe M-4169_FAM (5'-FAM-TTCTCTAGCAGTGGGACAGCCTGC-BHQ1-3'). Primers and probe were used at 10 pmol per reaction. RT-PCR was performed on a LightCycler 480 or LightCycler Pro instrument (Roche) under the following thermocycling conditions: reverse transcription at 50 °C for 30 min; initial denaturation at 95 °C for 15 min; followed by 40 cycles of denaturation at 95 °C for 15 s and annealing/extension at 56 °C for 1 min, with fluorescence acquisition at the end of each cycle.

GFP RNA corresponding to the NDV M gene was included as a positive control in each run. The positive control was kindly provided by P. Becher, University of Veterinary Medicine Hannover.

For detection of a second target, the fusion (F) gene, a primer–probe mixture was used as previously described [5]. The assay included the forward primer F+4829 (5′-GGTGAGTCTATCCGGARGATACAAG-3′) and the reverse primer F−4939 (5′-AGCTGTTGCAACCCCAAG-3′). Detection was performed using a set of genotype-specific hydrolysis probes: F (VFP-1)-4894_FAM (5′-AAGCGTTTCTGTCTCCTTCCTCCA-3′), F+4870_XIV_FAM (5′-TGGAGGAAGACGACGGAAACGTTT-3′), and F−4894_VII_FAM (5′-AARCGTTTTTGTCTCCTTCCTCCG-3′). In addition, the forward primer F+4835_48F (5′-TCCATCCGCAAGATCCAAGG-3′) was included as described. Primers and probes were used at concentrations and under cycling conditions as previously reported [5].

Amplicon RT-PCR

Reverse transcription PCR was performed using SuperScript IV reverse transcriptase (Thermo Fisher Scientific, USA), according to the manufacturer's instructions, targeting the viral RNA genome with the oligonucleotide primer A2-Fwd-1811 (ACGGGTAGAAGAGAGACA). Per sample, 1 – 20 ng total RNA input was used. Reverse transcription was carried out at 50 °C for 10 min, followed by enzyme inactivation at 80 °C for 10 min. Resulting cDNA was amplified by PCR using the NEBNext® Ultra™ II Q5® kit (New England Biolabs, USA) with the oligonucleotide primers A2-Fwd-1984 (GCGGAGACTATTGGGAAGA) and A2-Rev-5217 (AGGGGAAGTGATTTGTGG). Initial denaturation (30''/ 98°C) was followed by 32 cycles of 10''/98°C, 30''/62°C, 90''/72°C and a final extension of 2'/72°C. The resulting amplicons were of the expected sizes (3,234 bp).

Metatranscriptomic library preparation and sequencing

Untargeted high-throughput short-read sequencing was performed on total RNA isolated from brain biopsy tissue, post-mortem brain tissue, and urine. Following DNaseI treatment (Thermo Fisher Scientific, USA), RNA was used for library preparation via the SMARTer Stranded Total RNA-Seq Kit v2 - Pico Input Mammalian (Takara Bio Europe, Saint-Germain-en-Laye, France). Libraries were sequenced

on an Element Biosciences AVITI (2x75 bp and 2x150 bp, PE protocol) and an Illumina NextSeq 2000 instrument (2x 150 cycles, PE protocol).

Metatranscriptomic short-read analysis

Samples were analyzed as recently described using an in-house pathogen detection pipeline combining mapping, k-mer, and *de novo* assembly approaches. Raw sequencing reads were first assessed for quality using FastQC (v0.12.1) prior to adapter and quality trimming with trim_galore (v0.6.10). Trimming removed short reads, low-quality bases, reads with excessive ambiguous nucleotides (N), and residual Illumina adapter sequences. The filtered reads were subsequently aligned to the human reference genome GRCh38 (ENSEMBL) using BWA-mem2 (v2.2.1) [13]. Only high-confidence alignments, defined as uniquely mapped reads with a mapping quality score (MAPQ) of 60, were retained using sambamba (v1.0.0) [12]. Reads that did not map to the human genome were extracted from the original FASTQ files using BBTools [3] and subsequently aligned to the NCBI Viral RefSeq database using BWA-mem2. To eliminate spurious hits, alignment scores were filtered for those in which at least 5% of the viral genome exhibited a minimal read coverage of 10, and remaining hits were ranked by the total number of reads mapped to individual species.

In parallel, host-subtracted reads were subjected to k-mer-based taxonomic classification with Kraken2 (v2.1.3) [14], followed by abundance estimation using Bracken [7], and to *de novo* assembly contig-based pathogen detection using the previously described DAMIAN [1] pipeline.

For identification and abundance estimation of SNPs, metagenomic reads were mapped to AOaV-1 isolate DOZA007M22 (GenBank accession OQ745938) using BWA-mem2, followed by SNP detection using the bcftools (v1.17) mpileup and call commands with default conditions. Resulting vcf files were filtered for variant calls with quality scores above 50 for the analysis of major and minor variants shown in Figure 1A. For metagenomic consensus sequences, variant calls were further filtered for those with allele frequencies of at least 0.9, followed by consensus creation with the bcftools consensus command.

Long-read amplicon library preparation and sequencing

Long-range PCR amplicons were purified using NucleoMag NGS clean-up beads (Macherey-Nagel, Germany) at a 0.6× bead-to-sample ratio and quantified using the Qubit dsDNA High Sensitivity assay (Thermo Fisher Scientific, USA). Purified amplicons were subjected to end repair and dA-tailing using the NEBNext Ultra II End Repair/dA-Tailing Module (New England Biolabs, USA), followed by ligation of sequencing adapters using the Ligation Sequencing Kit with native barcoding (SQK-NBD114-24; Oxford Nanopore Technologies, UK), according to the manufacturer's instructions.

Sequencing was performed on a PromethION 2 Solo device using R10.4.1 flow cells (PromethION). Real-time base calling was performed using Dorado 7.6.7 (Oxford Nanopore Technologies, UK) in high-accuracy mode. Reads were demultiplexed and quality-filtered prior to downstream analyses.

Haplotype reconstruction

Haplotypes were reconstructed by long-read sequencing of amplicons spanning nucleotides 1,984–5,217. Long reads were filtered and trimmed based on the presence of correct amplicon primer pairs at the corresponding end of the amplicon reads using cutadapt (v3.5.) [9]. Highest quality reads of each sample (50% of the trimmed/filtered reads) were then selected for haplotype detection using FiltLong (https://github.com/rrwick/Filtlong). Haplotypes within the respective sample were generated, filtered and combined based on similarity using strainline with --maxGD=0.001, --maxLD=0.0001 and --dsim=0.95 [8]. To eliminate indels resulting from nanopore sequencing errors, generated sequences were inspected for short indels (maximum length 2) residing within or next to homopolymeric stretches of at least 3 nucleotides. Corresponding indels supported by only a single haplotype were considered erroneous and corrected for downstream analyses. The corrected sequences were subsequently collapsed onto unique representatives for final haplotype assignment. Abundance estimations for individual haplotypes were made according to read counts reported by strainline.

For annotation and database submission, full-length genome sequences of urine-derived haplotype H0 (representative of the ancestral virus SMP60/HH/2025) as well as biopsy-derived haplotypes H1.1 and H2.1 were assembled from long-read data and flanking consensus sequences from metatranscriptomic short-read data. Partial genome sequences from long-read data were submitted for haplotypes recovered from post-mortem CNS samples (H1.2, H1.3, H1.4, H1.5, H2.2, and H2.3). GenBank accession numbers of annotated haplotype sequences are provided in the data availability statement.

Phylogenetic analysis

For family level analyses, complete genome nucleotide sequences of representative members of the family Paramyxoviridae were retrieved from GenBank and aligned together with haplotype H0 from the newly identified AOaV-1 isolate (SMP-H0/HH/2025). Representative paramyxovirus GenBank files used are: a) representative files paramyxoviridae, AF077761, AY741404, AY225110, EU403085, EU622637, AB005795, AF014953, NC_002200, NC_002728, NC_007454, NC_025412 and NC_001560; b) Newcastle disease virus and pigeon paramyxovirus-1 strains: PV958093.1, PV260999.1, PP297102.1, KT163263.1, KM374060.1, PP297098.1, MK469967.1, MG840657.1, KM374056.1, MW271783.1, JQ993431.1, OQ745932.1, OQ745937.1, OQ745938.1. For the analysis of viral quasi-species, we performed multiple sequence alignments among all haplotype sequences recovered by long-read sequencing. Multiple sequence alignment was performed using Clustal (ClustalW/Clustal Omega, default parameters). Phylogenetic relationships were inferred using the maximum-likelihood method based on a general time-reversible nucleotide substitution model with gamma-distributed rate variation among sites. Tree robustness was assessed by bootstrap resampling with 1000 replicates. The resulting phylogenetic tree was visualized and annotated using FigTree (version 1.4.4), with branch lengths scaled to nucleotide substitutions per site.

4. Supplementary tables S1-S3

Table S1: Chronology of Clinical Course and Microbiological Investigations

Timeline	Specimen /Event	Investigations and Results
Day 0	Admission; CSF	CSF culture negative; Gram stain negative (no bacteria but detection of leucocytes). CSF qPCR negative for HSV-1/2, VZV, CMV, EBV, BK virus, JC virus, Enterovirus (species A-D); *Haemophilus influenzae*, *Listeria monocytogenes*, *Neisseria meningitidis*, *Streptococcus agalactiae*, *Streptococcus pneumoniae*; *Cryptococcus neoformans*, *Aspergillus* spp; *Toxoplasma gondii*.
Day 6	CSF	CSF culture negative; Gram stain negative. CSF qPCR negative for HSV-1/2, VZV, HHV-6, enterovirus, West Nile virus; *H. influenzae*, *L monocytogenes*, *N. meningitidis*, *S. agalactiae*, *S. pneumoniae*; *C. neoformans*.
Day 7	Clinical course	Transfer to intensive care unit.
Day 11	CSF	CSF culture negative; Gram stain negative, NDV qPCR (M-gene) negative*.
Day 18	Tracheal secretion	NDV qPCR (M-gene) negative*.
Day 21	Brain biopsy	Histopathology: lymphocytic meningoencephalitis.
Day 27	Urine	NDV qPCR positive ct 32.6 (M-gene)*.<br>Positive for NDV by total RNA shotgun sequencing*;<br>Successful NDV haplotype recovery by long-read amplicon sequencing*.
Day 28	Metagenomics from brain biopsy	Metagenomic detection of NDV by unbiased total RNA shotgun sequencing;<br>NDV qPCR positive, Ct values 24.0 (F-gene) and 27.7 (M-gene);<br>Successful NDV haplotype recovery by long-read amplicon sequencing.
Day 33	CSF	NDV qPCR (F-gene and M-gene) negative.
Day 33	Plasma	NDV qPCR (F-gene and M-gene) negative.
Day 34	Plasma	qPCR negative for herpesviruses, CMV, EBV, VZV, HSV.
Day 40	Outcome	Patient deceased.
Day 41	Post-mortem brain tissue	NDV qPCR positive. ct values F-gene (M-gene): temporal cortex II 22.6 (25.9); temporal cortex I 21.9 (25.4); cerebellum 26.1 (26.4); occipital cortex II 22.3 (27.9); brainstem 24.8 (27.3); optic nerve 27.7 (30.4); occipital white matter 22.8 (27.9).<br>Successful NDV haplotype recovery from all tissue specimen by long-read amplicon sequencing
Day 42	Post-mortem tissue (non-CNS)	Testes, Heart, Urinary bladder, Spleen, Liver, Lungs, Jejunum, Kidneys NDV qPCR (F-gene) negative.

Day 0 denotes hospital admission. Dates refer to specimen collection or clinical events unless explicitly identified as dates of diagnostic results. *retrospectively tested after metagenomic Newcastle disease virus identification on Day 28; NDV: Newcastle disease virus; HSV: Herpes Simplex Virus; VZV: Varicella Zoster Virus; CMV: Cytomegalovirus; EBV: Epstein-Barr Virus; HHV-6: Human Herpes Virus 6

Table S2: Primary results reported by clinical metagenomic analysis platform

Pipeline	Kraken/Bracken	VDB/bwa-mem2	Damian
Method [a]	k-mer	mapping	assembly
Host reads [b]	55,200,209	54,723,413	86,465,184
- % of all reads	59.56%	59.04%	93.29%
Non-host reads [c]	591,182	100,212	858,338
- % of all reads	0.64%	0.11%	0.93%
Species [d]	Orthoavulavirus javaense	Orthoavulavirus javaense	Orthoavulavirus javaense
Rank [e]	3	1	1
Assigned reads [f]	88,743	99,801	823,578
- % of all reads	0.10%	0.11%	0.89%
- % of non-host reads	15.01%	99.59%	95.95%

UMeRS analysis of 92,684,301 paired end reads from stereotactic cerebellar biopsy tissue by a clinical metagenomics platform [1, 6] combining three independent metagenomic pipelines (see text and methods for details).

[a] principal metagenomic detection method: k-mer matching (Kraken/Bracken) or read mapping (VDB/bwa-mem2) against a database of microbial or viral reference sequences, respectively, or *de novo* contig assembly followed by BLAST analysis against the NCBI nt database (Damian) [1].

[b] Number and percentage of reads assigned to the host (*Homo sapiens*) by each pipeline.

[c] Number and percentage of reads taxonomically assigned to other (non-host) organisms by each pipeline

[d] Name of highest ranking viral species reported by each pipeline.

[e] Rank of the viral hit among all hits reported by each pipeline (note that the first two hits reported by Kraken/Bracken were fungal species that could not be confirmed in downstream analyses).

[f] Total number and percentage of reads assigned to avian orthoavulavirus species.

Supplementary Table S3: De novo assembled Newcastle disease virus (avian orthoavulavirus 1) contigs identified in brain tissue by metagenomic sequencing

Contig ID	Contig Length (bp)	Mapped Reads (n)	Haplo-type(s) [a]	Blast Results [b]					
				Subject Accession	Subj. Length (bp)	e-Val	Query Cover (%)	Identity, (%)	Subject Region
102021	10,695	394,523	H1.1, H2.1	OQ745938.1	15,192	0.0	100	98.32	4,391 - 15,192
102029	3,232	354,357	H1.1, H2.1	OQ745938.1	15,192	0.0	100	97.52	57 - 3,286
102129	1,302	46,354	H2.1	OQ745937.1	15,192	0.0	100	91.40	3,188 - 4,489
102606	724	18,036	H1.1	OQ745938.1	15,192	0.0	100	90.06	3,262 - 3,985
102468	798	10,308	H1.1	OQ745938.1	15,192	0.0	100	86.47	3,188 - 3,985

De novo-assembled metagenomic contigs and associated blast results reported by the Damian [1] pipeline.

[a] retrospective assignment of haplotypes as identified by long-read amplicon sequencing. Contigs 102129, 102606 and 102468 correspond to hypermutated genome regions, whereas contigs 102029 and 102021 represent left and right flanking regions shared by both haplotypes.

[b] Results for highest scoring hit from alignment against the NCBI nr database

5. Supplementary Figures

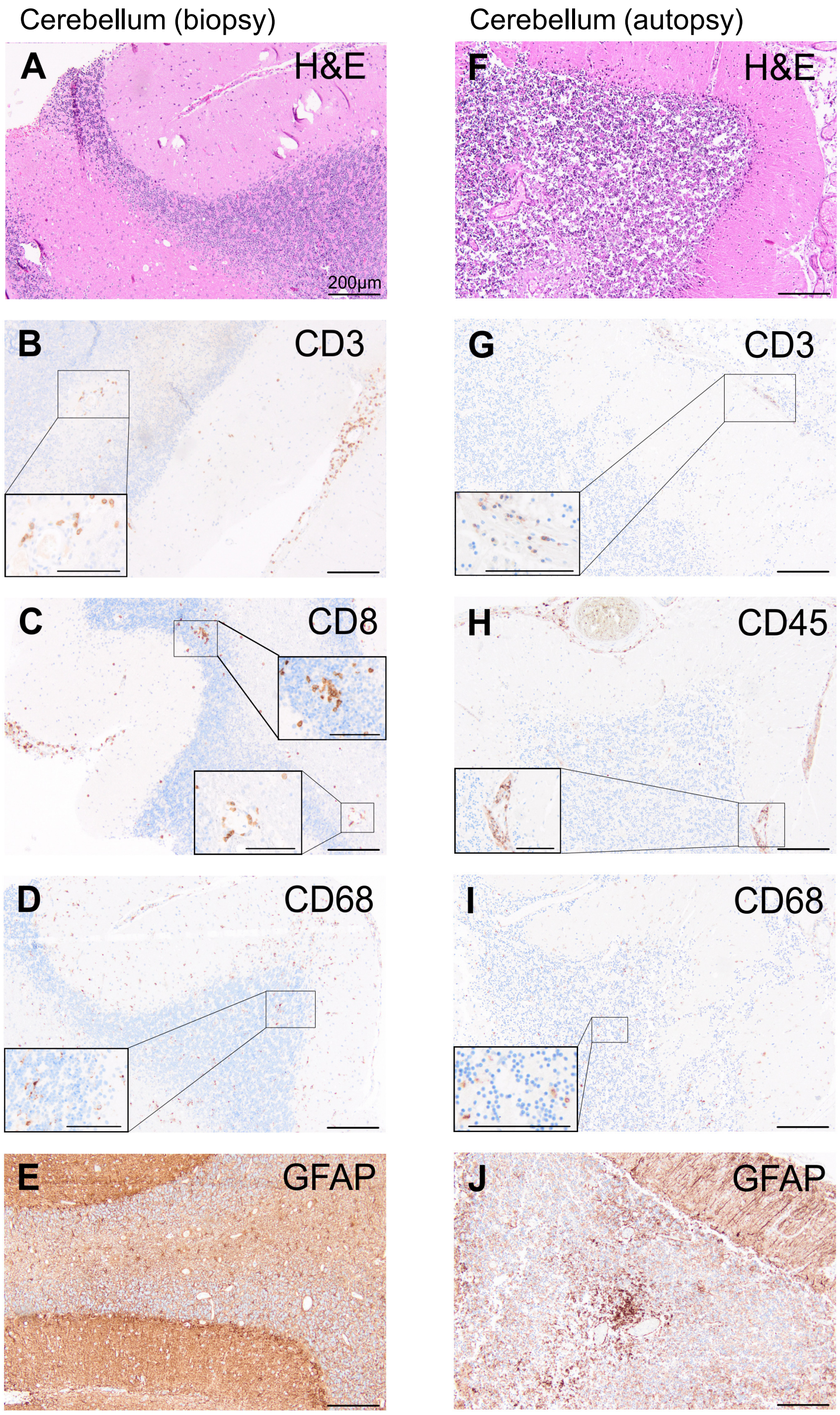

Supplementary Figure S1: Neuropathological findings in the cerebellum.

(A-E) Representative histopathological images of the cerebellar biopsy. The overview demonstrates preserved cerebellar histoarchitecture (A), with moderate T-cell infiltration in the leptomeninges and cerebellar parenchyma with pronounced perivascular accentuation. T cells are positive for CD3 (B) and CD8 (C). CD68 immunohistochemistry demonstrates moderate, diffuse microglial activation (D). Glial fibrillary acidic protein (GFAP) staining reveals reactive astrocytosis (E).

(F-J) Representative histopathological images of cerebellar tissue obtained at autopsy. The tissue sections show extensive autolysis with loosening and partial loss of the tissue architecture. The overall distribution of the inflammatory infiltrate remains comparable to that observed in the cerebellar biopsy (G-J). As expected, immunoreactivity is generally weaker in the autopsy tissue (G-J). CD45 staining is shown instead of CD8 (H), as the latter failed to yield a positive signal. Scale bars represent 200 µm in the overview panels (A-J) and 100 µm in all inset images.

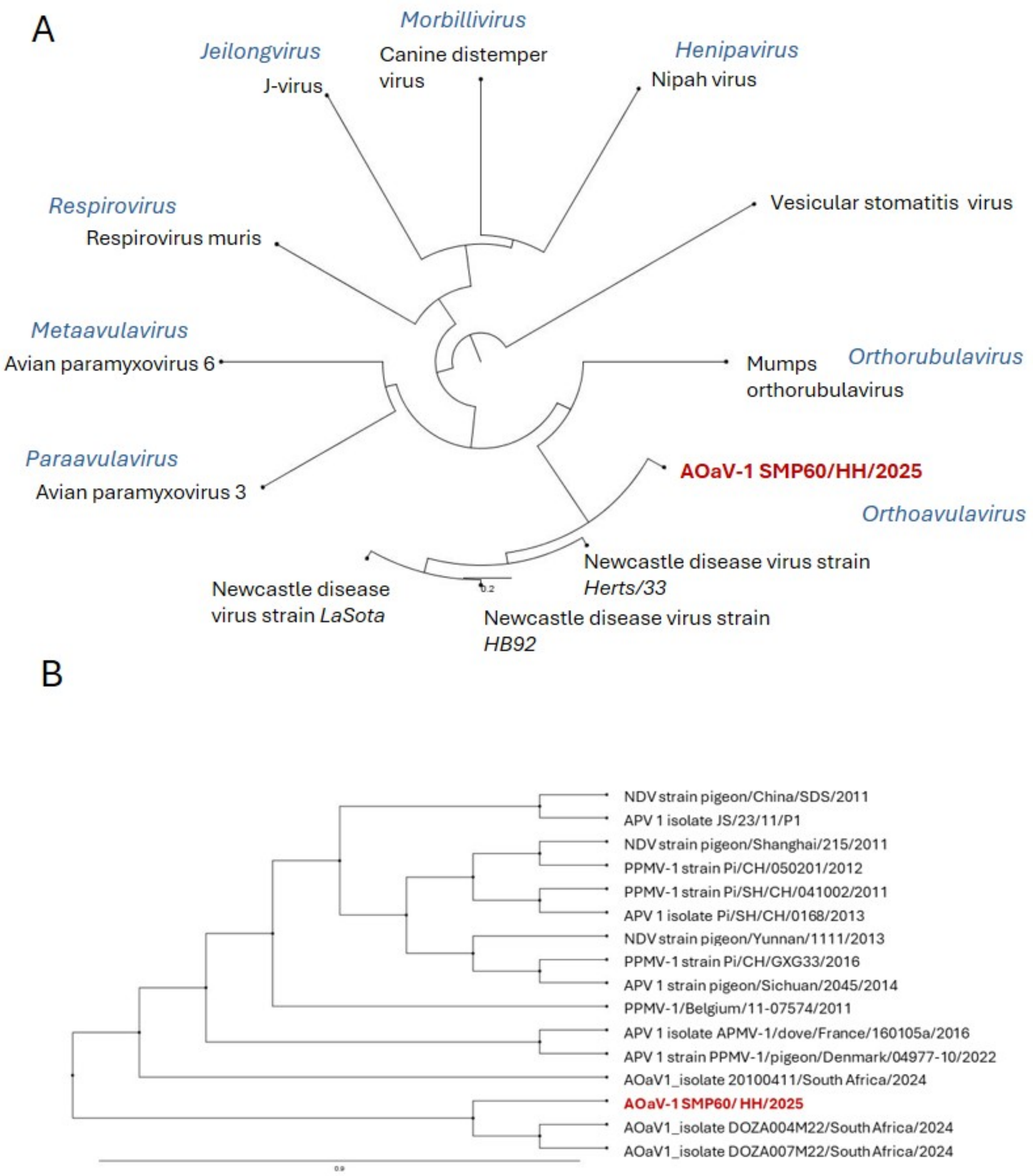

Supplementary Figure S2: Phylogenetic analysis of AOaV-1 SMP60/HH/2025.

(A) Circular maximum-likelihood phylogenetic tree inferred from complete genome nucleotide sequences of representative members of the order *Mononegavirales*, family *Paramyxoviridae* and *Vesicular stomatitis virus* NC_001560.1 as a representative of the family *Rhabdoviridae*. Major genera (in blue) are indicated, including *Orthorubulavirus* NC_002200.1, *Orthoavulavirus* AF077761.1/AY225110.1/AY741404.1/AOaV1, *Paraavulavirus* EU403085.1, *Metaavulavirus* EU622637.2, *Respirovirus* AB005795.1, *Jeilongvirus* NC_007454.1*, Morbillivirus* AF014953.1, and *Henipavirus* NC_002728.1. The newly identified avian orthoavulavirus type 1 isolate, AOaV-1 SMP60/HH/2025, is highlighted in red. Branch lengths are proportional to nucleotide substitutions per site. Complete genome sequences representing the type or prototype species of each recognized genus within the family *Paramyxoviridae*, with emphasis on avian paramyxoviruses, were retrieved from GenBank and included to ensure robust family-level phylogenetic placement of the newly identified avian isolate.

(B) Maximum-likelihood phylogeny inferred from complete genome nucleotide sequences of avian orthoavulavirus type 1 (AOaV-1), including representative Newcastle disease virus (NDV) and pigeonparamyxovirus-1 strains, GenBank accession numbers: OQ745932.1; OQ745937.1; OQ745938.1; PV958093.1; PV260999.1; JX901123.1; MG840657.1; MK469970.1; KM374056.1; KT163263.1; PP297100.1; KM374060.1; JQ979176.1; MW271783. The newly identified isolate, AOaV-1 SMP60/HH/2025, is shown in red. Scale bar indicates nucleotide substitutions per site. Phylogenetic analysis demonstrated that the isolate clustered most closely with pigeon paramyxovirus type 1 strains from southern Africa, in keeping with the patient's recent travel history.

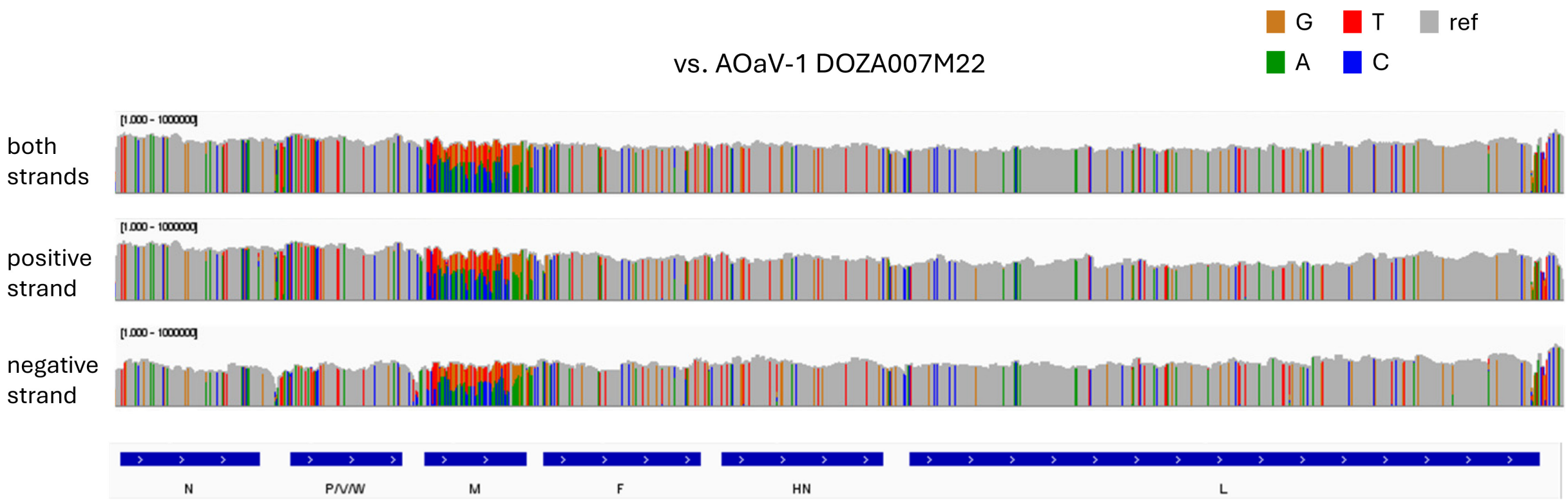

Supplementary Figure S3: Coverage plot of metagenomic reads mapped to AOaV-1 isolate DOZA007M22.

Screenshots from Integrated Genome Viewer coverage plots of metagenomic reads mapped to both strands, or positive (anti-genomic) or negative (genomic) strand of AOaV-1 isolate DOZA007M22 (GenBank accession OQ745938). Coverage is shown on a logarithmic scale (y-axis data range 1 to 1,000,000). Median coverage values are 3,195, 1,329.5 and 1,201 for both strands, positive strand or negative strand, respectively. Nucleotide allele frequencies are shown at variant loci according to the color code at the top, whereas invariant loci identical to the reference sequence are shown in grey.

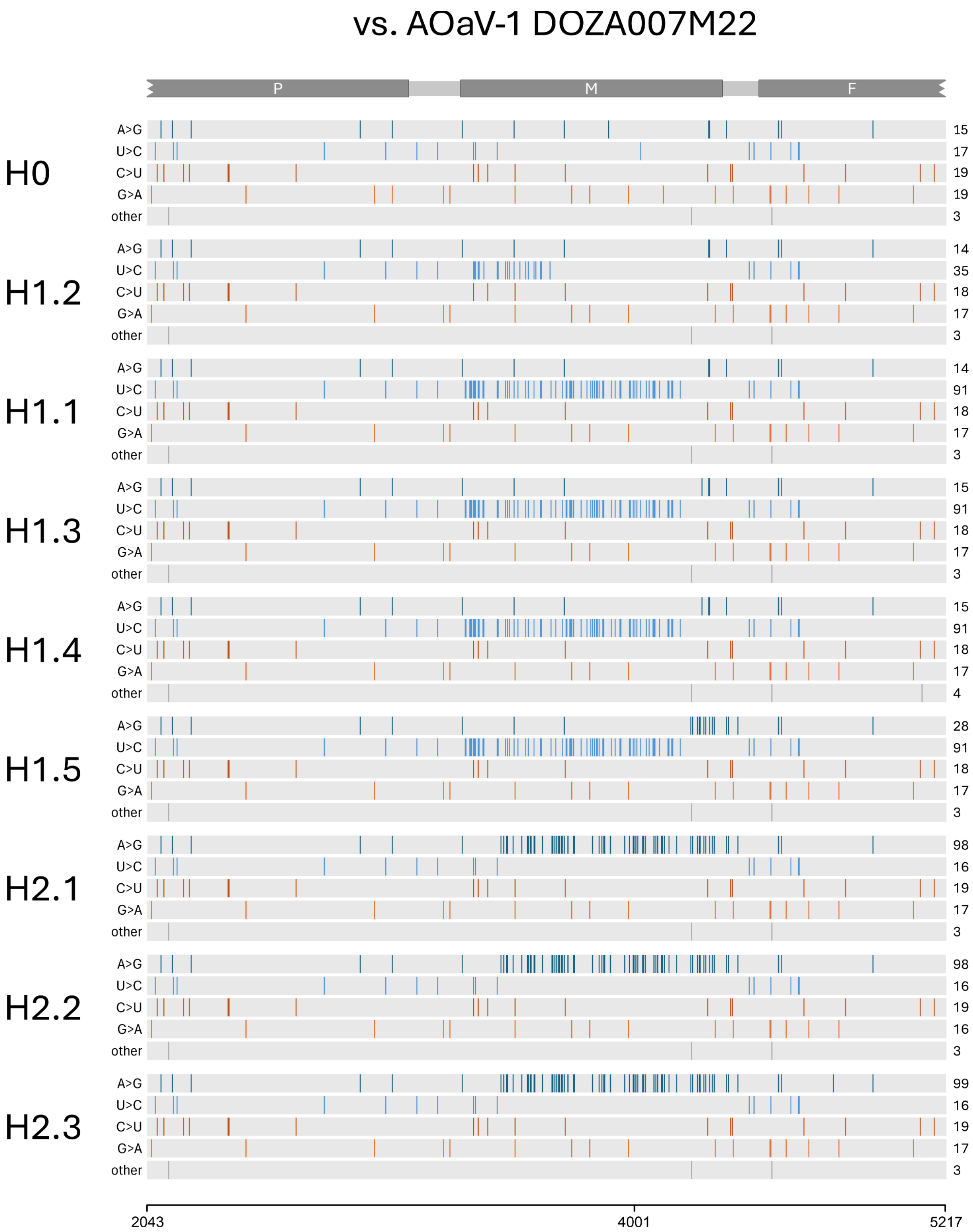

Supplementary Figure S4: Nucleotide substitutions in viral AOaV-1 SMP60/HH/2025 haplotypes relative to AOaV-1 isolate DOZA007M22.

Each bar graph depicts positions of indicated nucleotide substitutions in individual haplotype amplicon sequences aligned to AOaV-1 isolate DOZA007M22 (GenBank accession OQ745938). The total number of substitutions in the aligned region is shown to the right.

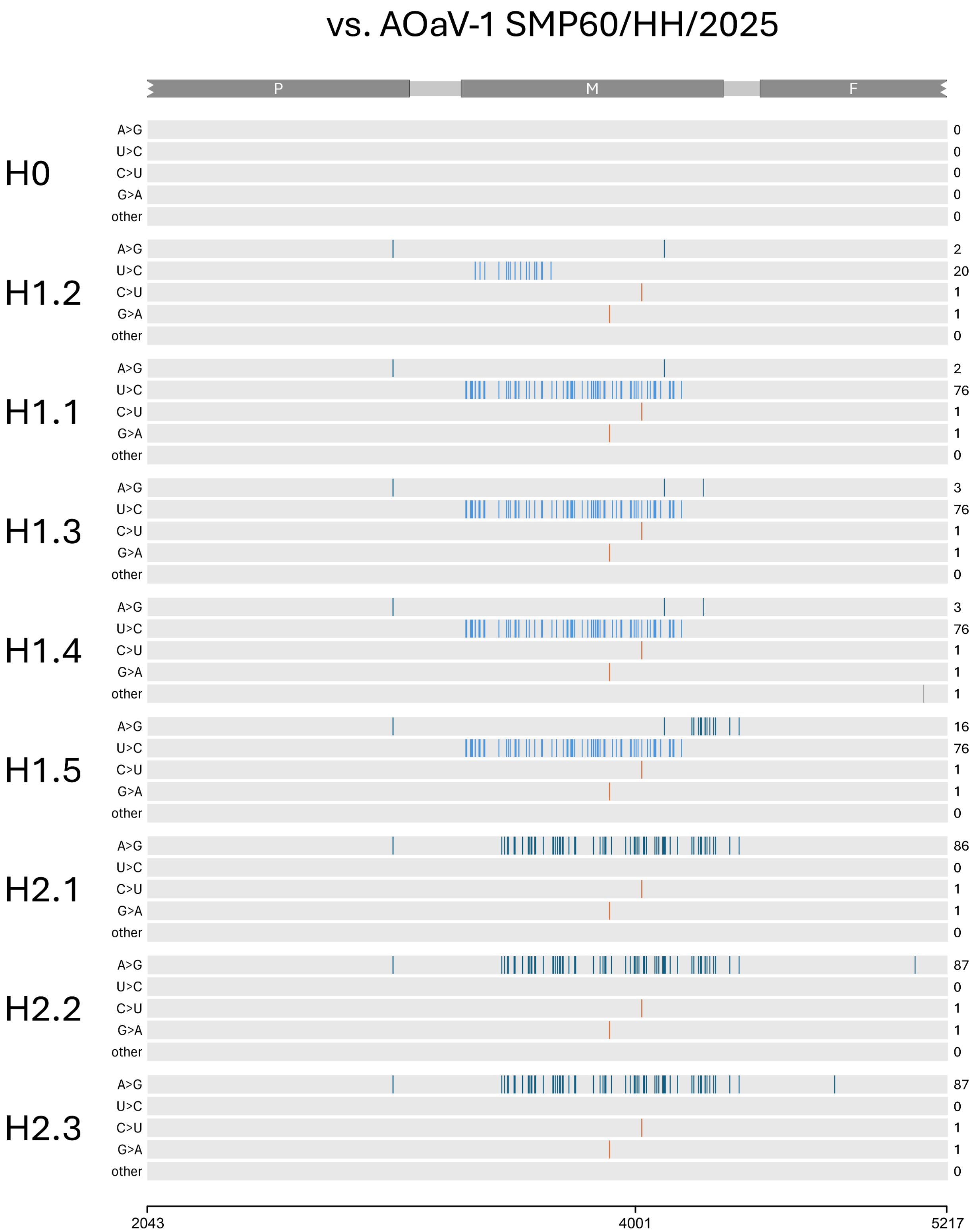

Supplementary Figure S5: Nucleotide substitutions in viral AOaV-1 SMP60/HH/2025 haplotypes relative to AOaV-1 SMP60/HH/2025 haplotype H0

Bars depict positions of indicated nucleotide substitutions in individual haplotype long-read amplicon sequences aligned to the ancestral haplotype H0 (AOaV-1 SMP60/HH/2025). The total number of substitutions in the aligned region is shown to the right.

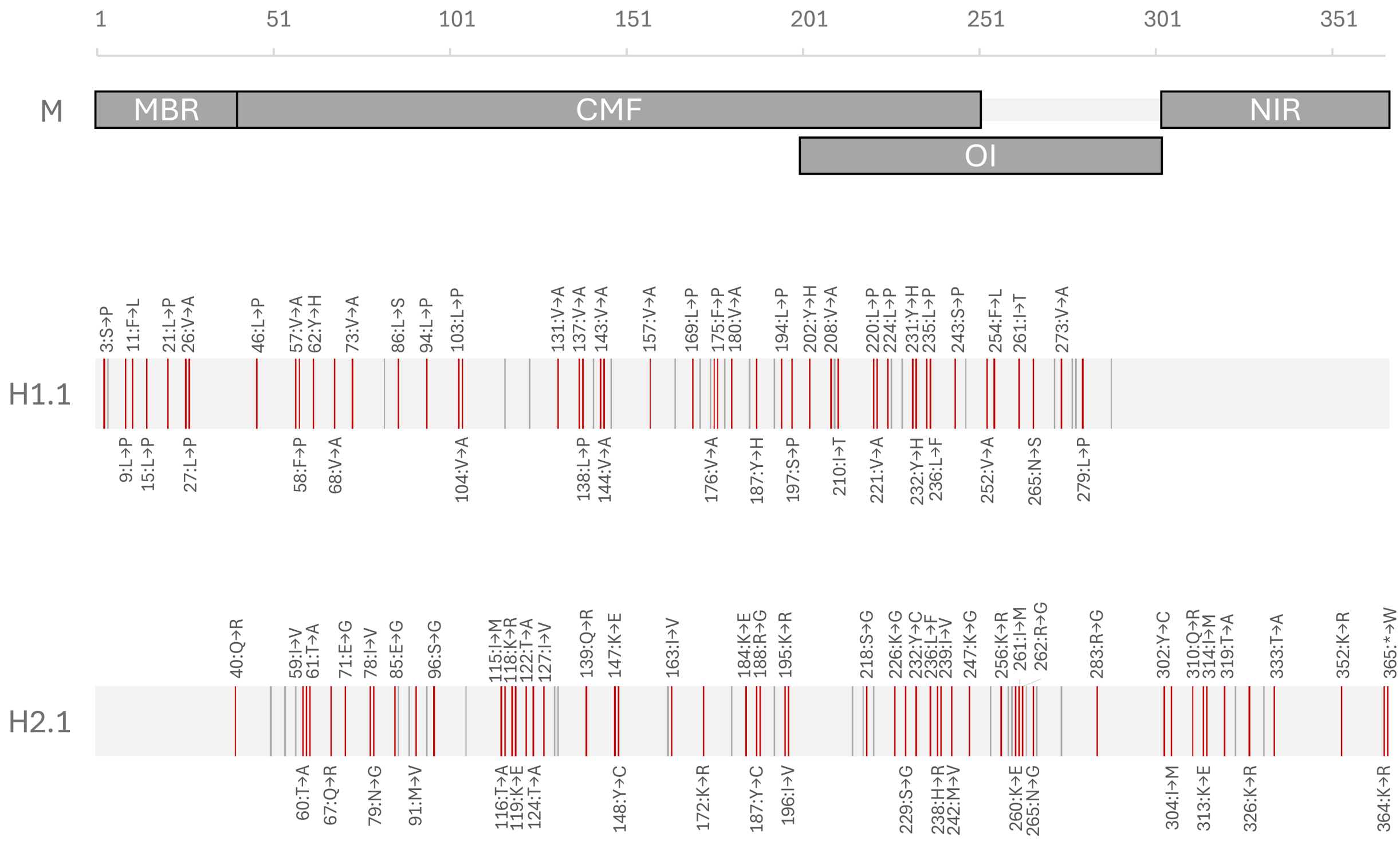

Supplementary Figure S6: Codon changes and amino acid substitutions in hypermutated AOaV-1 SMP60/HH/2025 M proteins

Shown are synonymous and non-synonymous codon mutations (grey and red bars, respectively) in the hypermutated M proteins encoded by viral haplotypes H1.1 and H2.1, relative to the ancestral haplotype H0 (AOaV-1 SMP60/HH/2025). Individual amino acid (aa) positions and substitutions are indicated above or below individual non-synonymously mutated codons. Note that changes in haplotype H2.1 include a mutation of the stop codon to a tryptophan-encoding codon (TAA to TGG), resulting in a M-protein with an additional 27 aa (sequence: WVASLRLRATRFPELSRHQIMIYLDCL) encoded by the intergenic region between the M- and F-genes. The map at the top shows functional regions or domains in the viral matrix protein (*MBR*: N-terminal basic / membrane-binding region, *CMF*: Core M protein fold, *OI*: Oligomerization interface, *NIR*: C-terminal regulatory / nuclear interaction region).

6. References

1 Alawi M, Burkhardt L, Indenbirken D, Reumann K, Christopeit M, Kroger N, Lutgehetmann M, Aepfelbacher M, Fischer N, Grundhoff A (2019) DAMIAN: an open source bioinformatics tool for fast, systematic and cohort based analysis of microorganisms in diagnostic samples. Sci Rep 9: 16841 Doi 10.1038/s41598-019-52881-4

2 Alexander DJ (2000) Newcastle disease and other avian paramyxoviruses. Rev Sci Tech 19: 443-462 Doi 10.20506/rst.19.2.1231

3 Bushnell B (2014) BBMap: A Fast, Accurate, Splice-Aware Aligner. Lawrence Berkeley National Laboratory LBNL Report #: LBNL-7065E City

4 Cathomen T, Mrkic B, Spehner D, Drillien R, Naef R, Pavlovic J, Aguzzi A, Billeter MA, Cattaneo R (1998) A matrix-less measles virus is infectious and elicits extensive cell fusion: consequences for propagation in the brain. EMBO J 17: 3899-3908 Doi 10.1093/emboj/17.14.3899

5 Kuiken T, Breitbart M, Beer M, Grund C, Hoper D, van den Hoogen B, Kerkhoffs JH, Kroes ACM, Rosario K, van Run Pet al (2018) Zoonotic Infection With Pigeon Paramyxovirus Type 1 Linked to Fatal Pneumonia. J Infect Dis 218: 1037-1044 Doi 10.1093/infdis/jiy036

6 Lopez-Labrador FX, Huber M, Sidorov IA, Brown JR, Cuypers L, Laenen L, Vanmechelen B, Maes P, Fischer N, Pichler Iet al (2024) Multicenter benchmarking of short and long read wet lab protocols for clinical viral metagenomics. J Clin Virol 173: 105695 Doi 10.1016/j.jcv.2024.105695

7 Lu J, Breitwieser FP, Thielen P, Salzberg SL (2017) Bracken: estimating species abundance in metagenomics data. PeerJ Comput Sci 3: Doi 10.7717/peerj-cs.104

8 Luo X, Kang X, Schonhuth A (2022) Strainline: full-length de novo viral haplotype reconstruction from noisy long reads. Genome Biol 23: 29 Doi 10.1186/s13059-021-02587-6

9 Martin M (2011) Cutadapt removes adapter sequences from high-throughput sequencing reads. 2011 17: 3 Doi 10.14806/ej.17.1.200

10 Patterson JB, Cornu TI, Redwine J, Dales S, Lewicki H, Holz A, Thomas D, Billeter MA, Oldstone MB (2001) Evidence that the hypermutated M protein of a subacute sclerosing panencephalitis measles virus actively contributes to the chronic progressive CNS disease. Virology 291: 215-225 Doi 10.1006/viro.2001.1182

11 Raab MS, Weinhold N, Kortum KM, Kronke J, Fenk R, Weisel K, Podola L, Bertsch U, Brobeil A, Mersi Jet al (2026) Teclistamab-based induction treatment in transplant-eligible, newly diagnosed multiple myeloma: a phase 2 trial. Nat Med 32: 2440-2448 Doi 10.1038/s41591-026-04471-x

12 Tarasov A, Vilella AJ, Cuppen E, Nijman IJ, Prins P (2015) Sambamba: fast processing of NGS alignment formats. Bioinformatics 31: 2032-2034 Doi 10.1093/bioinformatics/btv098

13 Vasimuddin M, Misra S, Li H, Aluru S (2019) Efficient Architecture-Aware Acceleration of BWA-MEM for Multicore Systems. 2019 IEEE International Parallel and Distributed Processing Symposium (IPDPS). IEEE Computer Society, City, pp 314-324

14 Wood DE, Lu J, Langmead B (2019) Improved metagenomic analysis with Kraken 2. Genome Biol 20: 257 Doi 10.1186/s13059-019-1891-0

7. Other information

Author contribution statement:

Conceptualization: C.K., C.S., K.W., T.M., E.F., M.L., N.F., A.G.

Investigation: C.K., C.S., L.L., R.K., W.A., M.A.B., S.K., L.R., N.W., M.R., J.H., A.P., D.H., K.R., A.R., P.B., F.F., E.S., S.K., A.N., B.O., M.D.

Formal analysis: C.K., C.S., T.G., A.R., M.L., N.F., A.G.

Resources: C.K., C.S., T.G., M.L., A.G., N.F.

Writing – original draft: C.K., C.S., K.W., A.G., N.F.

Funding acquisition: A.G., M.F., M.L., N.F.

All authors critically reviewed the manuscript, approved the final version, and agree to be accountable for all aspects of the work.

Data availability statement:

All relevant short-read and long-read sequencing data have been deposited in the European Nucleotide Archive (ENA) under accession PRJEB111208. For haplotypes H0, H1.1, and H2.2, complete genome sequences reconstructed from short-read shotgun sequencing and long-

read amplicon sequencing data have been deposited in GenBank under accession numbers QB284243, QB284244, and QB284249, respectively. For haplotypes H1.2, H1.3, H1.4, H1.5, H2.1, and H2.2, partial genome sequences obtained from long-read amplicon sequencing have been deposited in GenBank under accession numbers QB284245, QB284246, QB284247, QB284248, QB284250, and QB284251, respectively.

Acknowledgements:

We thank Marisa Schmied, Claudia Schmidt and Dominik Nörz for technical assistance.

This work was supported by the Deutsche Forschungsgemeinschaft (DFG, German Research Foundation) within the SFB 1648/1 [2024] – Project-ID 512741711 and by the Federal Ministry of Education and Research (BMBF) through NUM-SAR and NATON within NUM (01KX2524).

L. L. received support by the German Cancer Aid through a Mildred Scheel Postdoctoral Fellowship and the Mildred Scheel Nachwuchszentrum at University Medical Center Hamburg-Eppendorf. C.S. is supported by the German Cancer Aid through a Mildred Scheel Postdoctoral Fellowship.
